# Optimising the management of children with concomitant bladder dysfunction and behavioural disorders

**DOI:** 10.1007/s00787-022-02016-4

**Published:** 2022-06-29

**Authors:** Dilharan D. Eliezer, Christopher Lam, Angela Smith, John Mithran Coomarasamy, Naeem Samnakay, Malcolm R. Starkey, Aniruddh V. Deshpande

**Affiliations:** 1 John Hunter Children’s Hospital, New Lambton Heights, NSW Australia; 2 University of Newcastle, Newcastle, NSW Australia; 3 Hunter New England Library, New Lambton Heights, NSW Australia; 4 Department of Surgery, Perth Children’s Hospital, Nedlands, WA Australia; 5 Division of Surgery, Medical School, University of Western Australia, Crawley, WA Australia; 6 Hunter Medical Research Institute, New Lambton Heights, NSW Australia; 7 Department of Immunology and Pathology, Central Clinical School, Monash University, Melbourne, Australia; 8 Urology Unit, Department of Surgery, Department of Paediatric Surgery, The Children’s Hospital at Westmead, Locked Bag 4001, Westmead, NSW 2145 Australia; 9 Centre for Kidney Research, The Children’s Hospital at Westmead, Westmead, NSW Australia; 10 Auburn Hospital, NSW Auburn, Australia; 11 Nepean Hospital, NSW Kingswood, Australia

**Keywords:** Bladder dysfunction, Behavioural disorder, Urotherapy, ADHD, Pharmacotherapy

## Abstract

Bladder dysfunction and behavioural disorders in children are commonly concomitant; hence, it is difficult to treat each in isolation. Pharmacotherapy is common treatment for behavioural disorders, and these medications may have intended or unintended positive or negative bladder sequelae. This review identifies the literature regarding the effects of behavioural pharmacotherapy on bladder functioning and possible bladder management strategies in children with concomitant behaviour and bladder disorders to enable clinicians to better manage both conditions. A PROSPERO registered PRISMA-guided review of three major databases was performed. After an initial scoping study revealed significant heterogeneity, a narrative approach was undertaken to discuss the results of all relevant cases relating to children being treated with pharmacotherapy for behaviour disorders and outcomes related to bladder function. Studies were screened to identify those that described effects of commonly prescribed medications in children with behavioural disorders such as stimulants, alpha 2 agonists, tricyclic antidepressants (TCA), serotonin and noradrenergic reuptake inhibitors (SNRI), selective serotonin reuptake inhibitors (SSRI) and antipsychotics, and the findings and implications were summarised. The review identified 46 studies relevant to behavioural pharmacotherapy and bladder function (stimulants (*n* = 9), alpha 2 agonists (*n* = 2), TCAs (*n* = 7), SNRIs (*n* = 8), SSRIs (*n* = 8) and antipsychotics (*n* = 6). Six studies focused specifically on bladder management in children with behavioural disorders with concurrent behavioural pharmacotherapy. This review identifies useful factors that may assist clinicians with predicting unintended bladder effects following initiation of behavioural pharmacotherapy to facilitate the best approach to the treatment of bladder dysfunction in children with behavioural disorders. With this evidence, we have provided a useful decision-making algorithm to aide clinicians in the management of these dual pathologies.

**Supplementary Information:**

The online version contains supplementary material available at 10.1007/s00787-022-02016-4.

## Introduction

Behavioural disorders are highly prevalent and are one of the most commonly treated conditions of childhood [1, 2], and include Attention-deficit hyperactivity disorder (ADHD), Autism spectrum disorder (ASD), Oppositional defiance disorder (ODD) and Anxiety. Children with behavioural disorders, particularly autism, often experience delay in reaching developmental milestones, including difficulty in achieving day and night continence [3, 4]. Children with behavioural disorders are known to have increased incidence of urinary symptoms due to bladder dysfunction [5, 6]. It is estimated that 2–7% of school-aged children are diagnosed with ADHD [7] with lesser percentages for other disorders such as ASD, ODD and non-psychiatric anxiety-related disorders. In 2014, it was estimated that 314,000 (14%) children in Australia were affected by behavioural or mental health conditions [1].

Patients with a diagnosed behavioural disorder often have other disorders related to global development. Hence, there are a disproportionate number of children with behavioural disorders who also experience urinary incontinence [6, 8–12] in addition to other developmental problems such as faecal incontinence and learning delays. There is limited literature to clarify whether there is a cause–effect relationship between behavioural disorders and bladder dysfunction in children. Depending on the severity of their behavioural symptoms, children may require medications to manage the behavioural disorder. While the nature of the correlation between behavioural disorders and bladder dysfunction is unclear, it may be that they share a central cause with the effect of inattention or lack of focus and thus intentional behavioural disruption; leading to the theory that appropriate treatment of the behavioural disorder will assist in management of bladder dysfunction [13]. A wide range of medications, particularly those used for behavioural management, are known to have effects on the urinary bladder and urinary symptoms [13]. In ADHD management, it is estimated that 6.1% of children are prescribed medications and this number is 30.3% in Autism [14]. Often children will require multiple medications to manage their behaviours, in addition to non-pharmacological treatments. There is paucity of high-quality data on the impact of behavioural disorders and their associated medical therapies on bladder function and management and the efficacy of treatment of bladder dysfunction in children with comorbid behavioural disorders [15]. Therefore, in this wide-scoped, sensitive search enabled narrative review, we sought to identify pertinent literature related to the effects of behavioural pharmacotherapy on bladder functioning specifically and effective management strategies for bladder dysfunction in children with behavioural disorders.

## Methods

We performed a review using PRISMA (Preferred Reporting Items for Systematic reviews and Meta-Analyses) guidelines. The review protocol has been registered with PROSPERO (CRD42018094062). Relevant studies included in this review were case reports, case series, cohort studies, case-controlled studies, randomised controlled trials and quasi-randomised studies. An initial scoping search suggested that the available studies were too heterogenous to perform a conventional systematic review; hence, a narrative approach was undertaken.

Eligible studies had participants that were children aged 5–18 years with an identified behavioural disorder that affects functioning as per DSM Axis II disorders (i.e., pervasive behavioural disorders), including Autism Spectrum Disorder (ASD), Attention-deficit/hyperactivity disorder (ADHD), Oppositional Defiance Disorder (ODD), Conduct disorder and Anxiety-related disorders (without Depressive symptoms) that are often highly comorbid with other behavioural disorders. For eligibility, children had to be treated with a medication for management of their behavioural disorder. Comparison groups included children who do not take medication or children as cross-over comparison (pre or post medical treatment of behaviour), children taking placebo or children taking other medications. Systematic reviews were excluded from this analysis.

An electronic search using the EMBASE (1996–2018), MEDLINE (1996–2018), PUBMED and PSYCHINFO (2002–2018) databases to identify studies using appropriately selected search terms was performed (see appendix 1).

## Results

The search identified 2239 studies with 46 found to be eligible for full-text review (see appendix 2 for PRISMA search flowchart). The majority of studies were case reports (*n* = 15) and cohort studies (*n* = 15) with some case series (*n* = 8) and randomised controlled trials (*n* = 8).

Nine studies looked at the effect of stimulants on bladder dysfunction. Two studies looked at the use of alpha agonists. Seven studies examined the use of tricyclic antidepressants (TCAs) in bladder and behaviour. Eight studies have looked at Serotonin and noradrenergic reuptake inhibitors (SNRIs) for bladder treatment, four for nocturnal enuresis and four for stress incontinence. Eight studies looked at selective serotonin reuptake inhibitors (SSRIs) and bladder effects. Six studies looked at the effects of antipsychotics. The remaining six studies look at varied strategies for managing comorbid behaviour and bladder disorders.

### Medications and known bladder effects

Pharmacological agents are not without unintended side-effects and of particular interest to us is the effect of these agents (intended and unintended) on bladder function in children (see Table 1). We posed a series of clinically relevant questions to frame the discussion regarding the effects of these agents on bladder function.

**Table 1:** Common classes of medications used in behavioural disorder management, their role in bladder dysfunction, possible mechanisms of action, bladder effects and studies that provide evidence for effects of these medications

Medication	Behavioural indications	Bladder dysfunction indications	Bladder effects (pharmacological/physiological)	Clinical bladder effects	Overall bladder effects of medications and evidence
Stimulants	ADHD	Giggle incontinence	Attention/focus—prefrontal, reduce sleep arousal threshold with effectiveness for nocturnal enuresis	Resolves nocturnal enuresis in ADHD patients Giggle incontinence management Increases bladder capacity and voided volume	Primarily **positive** effects Cohort study [16, 17] NE case series [18–20]
Alpha 2 agonists	ADHD	Nil specified	Awareness/sleep arousal for nocturnal enuresis	There may be benefit in refractory nocturnal enuresis, 56.1% success in those that had failed desmopressin, alarms and anticholinergics though may not be in behaviour	Primarily **positive** effects [21]
SSRI	Anxiety/depression		5HT4 receptors—increasing detrusor tonicity and contractility—increased incontinence	Possible increased daytime incontinence except with fluoxetine, but small cohort studies suggest can ameliorate nocturnal enuresis	Primarily **mixed** effects Increased incontinence in [22, 23]] Changing to fluoxetine in case reports [24, 25] Treatment of nocturnal enuresis– [26]
SNRI	ADHD/anxiety/autism	Nocturnal enuresis Stress incontinence	Relaxes urinary bladder and increased urethral tone (Jost) via reduced uptake at Onuf’s nucleus within sacral spinal cord	Anti-enuretic effects, reduction in wet nights Improved stress incontinence symptoms in dose dependent manner	Primarily **positive** effects RCT for nocturnal enuresis [27] Case series nocturnal enuresis [28, 29]
TCA	Anxiety/depression/ADHD	Third line for nocturnal enuresis, some effect on overactive bladder	Focus and calm Resolves antipsychotic induced enuresis	Resolves nocturnal enuresis and some effect on overactivity Can counteract antipsychotic induced nocturnal enuresis	Primarily **positive** effects Overactive bladder [30, 31] Refractory nocturnal enuresis [29, 32, 33] Antipsychotic-induced nocturnal enuresis [34]
Antipsychotic	Autism/ODD/conduct disorder	Nil	Increased urethral tone via dopaminergic pathway and central micturition reflex,	Increased bladder relaxation and increased urethral tone–poor bladder emptying and retention/overflow	Primarily **negative** effects Case reports and case series–[17, 35–38]

Key: Bolded text highlights predominant net effect for the class of behavioural medications in the corresponding row

### Is there evidence that pharmacotherapy to primarily treat behavioural disorders may show benefit in aspects of bladder dysfunction?

#### Stimulants and effects on bladder function

Stimulants include such medications as methylphenidate, lisdexamfetamine, guanfacine and dextroamphetamine, with predominant use as a first-line medical therapy in ADHD. Two case series [18, 19] looked at the use of stimulants and resolution of nocturnal enuresis (NE) in children with ADHD. Williamson [18] reported on 3 children aged 9, 11 and 15 treated with 3 different stimulants and nocturnal enuresis resolved immediately after starting stimulants. In one child, symptoms recurred when stimulant treatment was ceased. [16] The hypothesis proposed by these authors was that stimulants might decrease sleep arousal threshold allowing children to awaken and exert continence control. Ferrara [20] also found nocturnal enuresis was adequately treated in six out of nine children with concurrent ADHD and nocturnal enuresis once methylphenidate was started or the dosage was increased. Bahali [19] reported a case of a 7-year-old girl where nocturnal enuresis resolved with stimulant treatment though atomoxetine, a noradrenaline reuptake inhibitor, prescribed to help moderate her ADHD symptoms.

In slightly contrary findings, Ferrara et al. [20] reported that nine children with ADHD and nocturnal enuresis responded well to increasing doses of methylphenidate alone to treat both disorders, while desmopressin was not effective in treating those children who were not medically treated for ADHD.

In summary, the studies supporting stimulants as a sole treatment for nocturnal enuresis are only case series with small numbers, but they do suggest that stimulants play a role. Bigger studies suggest a role for stimulants in helping responsiveness to other treatments for bladder disorders. In DUI, there was no evidence that stimulants alone improve symptoms, but there was good response to other bladder treatments for nocturnal enuresis, particularly desmopressin, especially if behaviours were pharmacologically treated with stimulants.

#### Clonidine and effects on bladder function

Clonidine is an alpha-2 agonist also used routinely to augment the treatment for ADHD as a second-line therapy. While its mechanism of action is unknown, it is thought to aide in improving attention and behaviour via increased sympathetic flow to the prefrontal cortex and possibly by improving the hypothalamus–pituitary axis in conjunction with stress hormones to improve attention and memory [39]. However, there is one study that focussed on the use of clonidine primarily on bladder management, and Ohtomo [21] found that clonidine was partially or completely effective in 56.1% of 148 children with nocturnal enuresis (both monosymptomatic and non-monosymptomatic) refractory to desmopressin, anticholinergics and alarm therapy. It is unclear if these children had comorbid behavioural disorders. A similar class of alpha agonist known as tizanidine (not used in behavioural management but has been trialled for nocturnal enuresis) found improvement in day- and night-time incontinence frequency as well as urgency symptoms and patient satisfaction [40].

In summary, there is limited evidence to support the role of alpha agonists in bladder dysfunction management, though the results from two studies appear to suggest a benefit from alpha 2 agonists in refractory nocturnal enuresis and diurnal incontinence.

#### Serotonin and noradrenergic reuptake inhibitors (SNRIs) and effects on bladder function

Serotonin and/or noradrenaline reuptake inhibitors (SNRIs) such as atomoxetine are used in the treatment of ADHD as well as anxiety/depression and autism, and there have been some anti-enuretic effects noted in studies [28]. In children with nocturnal enuresis and ADHD, the use of atomoxetine in combination with other medications was effective in a 4-child case series [30]. A randomised-control trial of 87 children with nocturnal enuresis and ADHD treated with atomoxetine (mean age 10.2 years) found a significant reduction in wet nights [27]. Atomoxetine was found to be superior to placebo in reducing the number of wet nights per week with 10 of 44 atomoxetine-treated and 17 of 43 placebo-treated children having ADHD. Ohtomo [41] found that atomoxetine was able to lead to statistically significant reductions in number of wet nights per month in 19 out of 24 children with combined ADHD and nocturnal enuresis who did not respond to alarm, anticholinergics or desmopressin, suggesting that behaviour preferably needs to be treated prior to bladder treatment. Reboxetine has been found to have good effect in combination with desmopressin for treating nocturnal enuresis without the cardiotoxicity of imipramine [29]. In this study, 10 (22.7%) of the test subjects and 17 (39.5%) of the placebo subjects were found to have ADHD [31]. Ghanizadeh [42] conducted an RCT with 43 children with comorbid ADHD and nocturnal enuresis, and found that treatment with methylphenidate and nortriptyline was superior to methylphenidate and placebo in reducing nocturnal enuresis events, but there was notable relapse of symptoms once treatment was withdrawn. Increased sphincter tone may have led to the reported urinary retention in one case of an 8-year-old child treated with atomoxetine with ADHD [43]. It is thought the mechanism of action of SNRIs is inhibiting reuptake of serotonin and norepinephrine in the presynaptic neuron in Onuf’s nucleus in the sacral spinal cord, which leads to relaxation of the urinary bladder and increased tone in the urethral sphincters [44].

In summary, the evidence for use of SNRIs in nocturnal enuresis and stress incontinence is strong, with several large RCTS and other small studies supporting its ability to improve symptoms with or without other bladder specific therapy.

### Is there evidence that pharmacotherapy to primarily treat behavioural disorders may have negative effects on aspects of bladder dysfunction?

#### Selective serotonin reuptake inhibitors (SSRIs) and effects on bladder function

Selective serotonin reuptake inhibitors (SSRIs) are predominantly for the management of anxiety/depression and can augment treatments for other behavioural disorders. They are thought to work on the bladder via the 5HT4 receptors which can potentiate the cholinergic receptors on the bladder leading to increased activity of the detrusor muscle [45, 46]. In a large population study of medication use and associated urinary incontinence in 21 735 Norwegian women [22], a strong association was found for developing incontinence from the use of SSRIs. This was also found in a Dutch study of 13,531 SSRI users demonstrating a risk ratio 1.75 for developing DUI when taking an SSRI [23]. In a case series of 3 children with anxiety and/or depression, sertraline lead to urinary symptoms with the change to fluoxetine resolving the symptoms in one case [24]. This change from sertraline to fluoxetine and resolution of urinary symptoms was also noted in a case reported by Maalouf [25]. Mahdavi-Zafarghandi [26] found a good response to sertraline in a cohort of 25 adolescents with nocturnal enuresis who failed treatment with desmopressin, with 12 children having full response to sertraline and 6 children having a partial response, though it is unclear if these children had comorbid behavioural disorders.

On the other hand, SSRIs were trialled as a treatment in a cohort of nine children with no significant anti-enuretic effect noted [47]. In a smaller case series of 3 children [48], SSRIs were found to lead to nocturnal enuresis. Kano [49] used urinary 17-hydroxycorticosteroids (17-OHCS) and 17-ketosteroid sulfates (17-KS-S) as a stress barometer, and found that it helped to predict which children with nocturnal enuresis would respond to fluvoxamine as a treatment for their nocturnal enuresis as those with higher levels of stress responded more favourably to fluvoxamine.

In summary, SSRIS appear to have negative effect on bladder function and increase the risk of nocturnal enuresis in large population studies, though some small studies have found it may help and changing to other SSRIs can reverse new-onset nocturnal enuresis. Hence, the evidence regarding SSRIs and bladder dysfunction remains unclear.

#### Antipsychotics and effects on bladder function

Antipsychotics are prescribed in just under a third of patients with autism but may also be prescribed in other disorders such as conduct disorder or ODD to manage aggressive behaviours [14]. There are many case reports and series on the impact of antipsychotics in children and bladder function. It is thought that antipsychotics work through the dopaminergic pathway to control the micturition reflex centrally [50, 51], and also, dopamine can activate alpha-1-adrenergic receptors to increase urethral sphincter tone and improve bladder emptying via these receptors [52] and 5-HT1A receptors in the bladder [35]. If too much bladder relaxation and increased urethral tone occur, micturition difficulty and even urinary retention may become an issue [53].

Cop [36] reported a case of urinary and faecal incontinence in a child with autism taking risperidone even at low doses of 0.25 mg/day with resolution of symptoms when the medication was ceased and no symptoms on olanzapine 5 mg/day. This was thought to be due to the alpha antagonistic effect of risperidone on the urethral and anal sphincters. Herguner [35] reported two similar cases in autistic children with nocturnal enuresis with symptoms resolving on cessation of risperidone and commencement of olanzapine. Dikshit [37] reported a case of conduct disorder treated with risperidone where nocturnal enuresis developed, but did not recur with the use of aripiprazole. Kantrowitz [38] reported three cases of risperidone-induced nocturnal enuresis. However, Bozkurt [17] noted aripiprazole induced nocturnal enuresis in a 16-year-old male with autism and epilepsy. However, in a 9-year-old boy with intellectual disability and nocturnal enuresis who previously been trialled on multiple antipsychotics, it was noted that starting aripiprazole leads to a cessation of symptoms [51].

In summary, there are many small case reports and series regarding antipsychotics and new onset of incontinence and nocturnal enuresis. While these are small studies, with reversal of symptoms with cessation of medications and overall poor association in all these studies, the evidence suggests a negative effect of antipsychotics on bladder function.

### Is there evidence that pharmacotherapy to primarily treat bladder dysfunction may have a beneficial effect on behavioural disorders?

#### Tricyclic antidepressants (TCAs) and effects on bladder function

Although tricyclic antidepressants (TCAs) are third-line therapy for nocturnal enuresis, there are reports that there are good behavioural side-effects in children who take imipramine, making them calmer and more focused [33, 54]. Similar effects were noted for those taking reboxetine [29]. It is thought that the bladder effects of TCAs are due to their direct anticholinergic effects on the bladder detrusor [32]. Desipramine has been shown to have some effectivity in both overactive bladder and in ADHD treatment [30, 31]. Imipramine has been used as a third-line therapy for nocturnal enuresis for some time. A study by Gepertz [33] found 64.6% of children with nocturnal enuresis refractory to other treatments responded well to imipramine and seven children that had concurrent ADHD had a good behavioural response, as well. Imipramine was found to be effective in resolving risperidone-induced nocturnal enuresis in one study [34].

In summary, there is large-volume good-quality evidence, including a Cochrane review [Caldwell 2016] for the use of TCA in managing nocturnal enuresis, and it is already a recommended third-line treatment. Our review suggests that there may be beneficial effects on behavioural disorders, as well.

### Is there evidence that pharmacotherapy to primarily treat bladder dysfunction may have a negative effect on behavioural disorders?

#### Desmopressin and anticholinergics

Desmopressin was found to aggravate ADHD symptoms with aggression noted to be a side-effect in 4% of 399 children in a Swedish trial [55]. This has been noted in other studies [56] and anticholinergics can also evoke hyperactivity and attention problems.

### Is there evidence that pre-treatment of behavioural disorders gives better results for bladder dysfunction treatment and what other factors impact effectiveness of non-pharmacological or first-line nocturnal enuresis treatment strategies in children concomitant behaviour and bladder issues?

#### Importance of pre-treating behaviour before bladder treatment and consideration of multimodal therapy

In a review of treatments for day-time and night-time wetting, von Gontard [15] suggests that behaviours need to be treated prior to instituting bladder treatments due to needing cooperation and compliance as well as the improved attention, focus and awareness on the treatment and its outcomes. In a conference abstract, Giuseppe [57] demonstrated a response to urotherapy in 6 children with autism; however, it is not specified if pharmacologically treated or what the response was. Hence, there are sparsity of data regarding the efficacy of urotherapy in this group and treatment response in general. An additional question is whether PMFT and timed voiding with watch to improve efficacy of urotherapy as demonstrated in other groups [58, 59], but further bladder behavioural treatments may not work in children with behavioural disorder given previous treatment response to bladder behavioural treatments such as alarm therapy [60]. A study by Eliezer et al. [61] suggested that urotherapy in a treated cohort of children with behavioural disorders, and improvement would be most noted in the children with day-time incontinence and bladder–bowel dysfunction particularly.

In many cases, simply treating ADHD with medications such as stimulants may resolve nocturnal enuresis [62]. In a study of 75 children with ADHD and incontinence (60 were on medication), all children had DUI, 87% had nocturnal enuresis and 88% had frequency [63]. Treatment was successful in 83.9% with a mixture of behavioural, anticholinergics and biofeedback. However, behavioural treatment alone was only effective (partial or complete resolution) in 11 of 56 (19.6%) of children who completed the study. The treatment success rate of monotherapy in nocturnal enuresis in this cohort is lower (23–60%) with a higher relapse rate than in children without behavioural or psychological disorders [64–68].

Chertin [69] performed a randomised-control trial with 54 children comparing imipramine to oxybutynin combined with desmopressin for treatment of nocturnal enuresis. In both groups, 23 of 27 children were on methylphenidate. They started all children on urotherapy and used adherence to this as an inclusion criteria for the study. While they stated that the response to this behavioural program was insignificant, the exact results were not reported. The study found that the combination treatment had a significant reduction in dysfunction voiding symptoms scoring and incidence of nocturnal enuresis which was superior to imipramine alone. Gor [70] performed a retrospective review of the efficacy of desmopressin and or anticholinergic in treating nocturnal enuresis in ADHD or Autism and found that they were as effective these children with behavioural disorders as in those without behavioural disorder.

#### Effectiveness of urotherapy

Urotherapy has good effect in day-time incontinence and moderate effect in nocturnal enuresis [62–70]. For over 50% of children with bladder bowel dysfunction, symptoms will settle with urotherapy which includes adequate fluid intake and constipation management. However, biofeedback or pharmacological agents may be required such as anticholinergics or alpha blockers [71]. In children with behavioural disorders, the ongoing use of antipsychotics in those with bladder bowel dysfunction and autism makes treatment difficult [72]. Additionally, it is unclear what the response to urotherapy would be without treating behaviour first, though a study by von Gontard [von Gontard 2019] [15] suggests that this would be difficult. A recent prospective study (DABBED) by several of this review’s authors [61] found urotherapy to be an effective treatment for children with medically treated behavioural disorders particularly day-time or bladder bowel dysfunction. However, response to urotherapy was poor in those with nocturnal enuresis, particularly non-monosymptomatic nocturnal enuresis, even after adjunctive therapies were added. In children with bladder disorders, bladder capacity and minimal voids/day predict good response to urotherapy. These results are not specific or have subgroup analysis data available for children with behavioural disorders and so generalisability is poor.

#### Effectiveness of bladder treatment adjuncts in behavioural disorders

Kovacevic [73] found that pharmacological intervention for nocturnal enuresis with desmopressin and/or anticholinergics was much more effective than bladder behavioural treatment alone in children with ADHD (*p* = 0.012). In addition, whether or not the child was on stimulant medications for ADHD did not affect the good response to pharmacological treatment for nocturnal enuresis. This suggests that early pharmacological treatment for nocturnal enuresis is more effective than behavioural modification in children with ADHD. A similar finding was reported by Yang [74] with the use of desmopressin in ADHD and non-ADHD children with nocturnal enuresis.

#### Compliance and adherence to treatment

Treatment of bladder dysfunction is more difficult in children with ADHD. In one study of 113 children with ADHD and nocturnal enuresis, ADHD children treated with alarms were only dry in 19% at 12 months compared to 66% of controls [60], and there was no difference if they were treated with medication (Desmopressin). However, non-compliance was noted in 38% of children with ADHD compared to 22% of controls [60]. Similarly day-time wetting resolved in 68% of children with ADHD compared to 91% of controls [60]. Once again, non-compliance was a major factor in timed voiding as a treatment with 48% of children with ADHD not adhering to treatment compared to 14% of those without ADHD. Adherence is a significant issue with adherence to urotherapy and medications found to be 70% for nocturnal enuresis in the general population [75] with the previously mentioned increased non-adherence rates in ADHD [60]. In paediatrics, adherence is complicated by the need for parental/guardian supervision and support [76].

## Discussion

We are able to list the beneficial effects and the possible clinical situations where different classes of medications may produce a positive impact on the bladder dysfunction (Table 2). The overall quality of the evidence is low, and hence, strong recommendations are not yet possible.

**Table 2:** Clinical therapy recommendations and risks in children with behavioural disorders

Clinical issue	Strong treatment effect (level of evidence^a^)	Weak treatment effect (level of evidence^a^)	Increased risk (level of evidence^a^)
Nocturnal enuresis management in setting of behavioural disorder pharmacotherapy	Alarms/desmopressin + stimulants (2b) TCAs especially for antipsychotic induced nocturnal enuresis and refractory nocturnal enuresis (4) Atomoxetine for refractory nocturnal enuresis (1b) Reboxetine + desmopressin (1b)	Urotherapy alone (2b) Desmopressin alone (2b) Alarms alone (2b) Alpha 2 agonists (2b) SSRI (very weak) (4)	Antipsychotics (risperidone) (4)
Urinary retention in setting of behavioural disorder pharmacotherapy	NA	NA	SNRIs- atomoxetine (4)^b^ Antipsychotics (4)
Day-time wetting in setting of behavioural disorder pharmacotherapy	NA	TCAs for overactive bladder (1a) Tizanidine (alpha 2 agonist) (1b)	SSRI (except fluoxetine) (2a)
True stress incontinence in setting of behavioural disorder pharmacotherapy^b^	NA	Urotherapy + SNRIs (1b)	NA

^a^Oxford centre for evidence-based medicine levels of evidence

^b^Very rare

From review of the evidence, our primary recommendation is that treatment of behavioural disorder prior to assessing and treating the bladder dysfunction is preferable. Evidence suggests that aspects such as poor attention and focus due to the behavioural disorder may be contributing to bladder dysfunction. In addition, some of the central mechanisms between behaviour and bladder may be shared such as brainstem inhibition in ADHD leading to inability to detect bladder signals or decreased arousal to a full bladder or reduced central adrenergic stimulation [77, 78]. Hence, the untreated behavioural disorder will likely negatively impact on adherence and compliance with treatment of bladder dysfunction and thus should be addressed in the first instance.

A number of findings from this review summarised in Table 2 may help with treatment decisions for children with comorbid behavioural disorders and bladder dysfunction. An understanding of the effects of the behavioural medications on bladder functioning is important to help predict treatments response and potential bladder effects. For example, autistic children started on risperidone who subsequently develop urinary incontinence may do better when switched to olanzapine to manage both behavioural and bladder symptoms. Children with ADHD and nocturnal enuresis may benefit from combination methylphenidate and atomoxetine treatment; though one should treat the behaviour disorder first, as incontinence drops to similar rates as the general population, and nocturnal enuresis may self-resolve.

In children with behavioural disorders, particularly ADHD, alarm training for nocturnal enuresis may be difficult despite adequate treatment of behaviour disorder. In children with anxiety, psychotherapy for management of both behaviour and bladder appears to be highly effective, though medications may be needed for refractory bladder symptoms. Methylphenidate can resolve nocturnal enuresis in children with ADHD when the ADHD is well controlled. Clonidine may be effective in nocturnal enuresis refractory to alarms, desmopressin and anticholinergics in the paediatric population. SSRIs do not appear to help with urinary symptoms unless they are stress-induced such as in children with anxiety. In this group, fluoxetine appears to be the safest of all the options and resolves unintended bladder symptoms caused by other SSRIs. SNRIs such as atomoxetine are very useful for both behavioural disorder and bladder dysfunction treatment, and may be an important therapy for combined symptoms, particularly ADHD and nocturnal enuresis. TCAs are useful in nocturnal enuresis as third-line treatment but also may have positive effects on children with behavioural symptoms. They also can counteract bladder symptoms that are induced by antipsychotics.

**Fig. 1:**
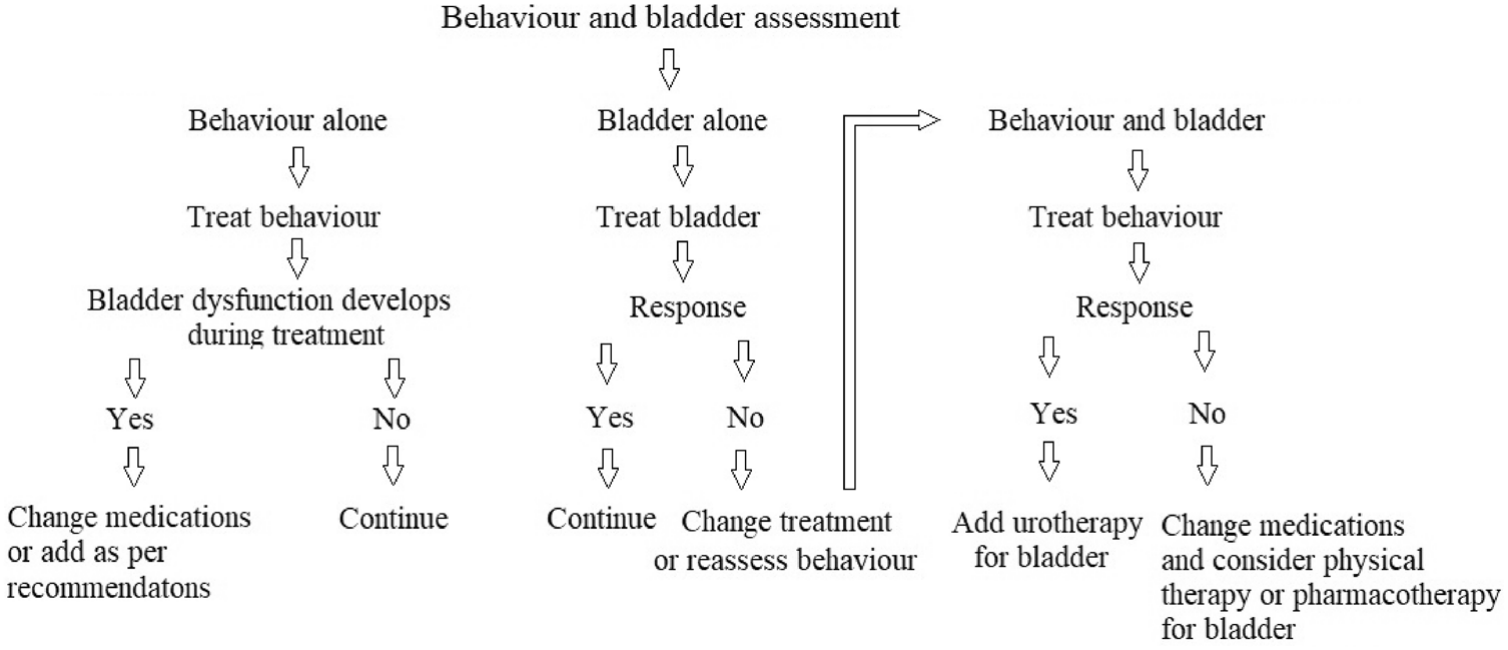
Proposed treatment algorithm for managing comorbid behaviour and bladder dysfunction

From this review, we propose a simple decision-making algorithm to optimise the treatment of children with behaviour and bladder dysfunction (Fig. 1). Children with well-treated behavioural disorders, response to bladder interventions may be similar to the general population, but developmental delay should be expected, particularly in children with ASD.

### Areas for further research

This review has elicited several areas where further research is required. In terms of pharmacological effects, the studies looking at the effect of antipsychotics are very small and larger cohort studies are required to verify this evidence; perhaps best achieved by systematic review through synthesis of available evidence. Alpha agonists have only been studied for their bladder effects, not in children with comorbid behavioural disorder and this effect in children with behavioural disorders requires further assessment.

In terms of treatment of comorbid problems, the evidence is very limited and studies looking at specific behavioural conditions and comorbid bladder dysfunction and the specific bladder treatments are required across all behaviours and treatments. An important consideration for developmental paediatricians would be if bladder dysfunction should be a criteria for pharmacotherapy in children with mild ADHD who would normally not require medication, and similarly with children with anxiety and concurrent bladder dysfunction, two domains where randomised controlled trials may be designed and implemented.

## Conclusions

This review demonstrates an important link between behavioural pharmacological agents and bladder function, especially in children with behavioural disorders. Overall, the evidence suggests that behavioural treatments such as stimulants, alpha 2 agonists and SNRIs have positive effects on the bladder. Certain SSRIs in specific instances can ameliorate bladder symptoms, but in other instances can worsen them. Antipsychotics have overwhelmingly negative effects on the bladder, though some are worse than others. Additionally, certain treatments specific to bladder may or may not work effectively in children with behavioural disorders; behavioural treatments such as alarm training working poorly in these children and medications such as anticholinergics potentially worsening behaviour. These demonstrated effects on bladder function can help clinicians choose or help refrain from groups of medications, depending on the nature and severity of underlying bladder dysfunction in a given child with behavioural disorder(s).

### Supplementary Information

Below is the link to the electronic supplementary material.

Supplementary file1 (DOC 43 kb)

Supplementary file2 (DOCX 35 kb)
