# Supplementary material for: Optimising the management of children with concomitant bladder dysfunction and behavioural disorders
Source: Eur Child Adolesc Psychiatry. 2022 Jun 29;32(10):1989–99. doi: 10.1007/s00787-022-02016-4 (PMC10533605; doi:10.1007/s00787-022-02016-4)
Supplement: Supplementary file 1 — Supplementary file1 (DOC 43 kb) [file 787_2022_2016_MOESM1_ESM.doc]

Database: Ovid MEDLINE(R) and Epub Ahead of Print, In-Process & Other Non-Indexed Citations and Daily

Search Strategy:

--------------------------------------------------------------------------------

1 exp Anxiety Disorders/ (75002)

2 Anxiety/ (71993)

3 exp "Attention deficit and disruptive behavior disorders"/ or Child behavior disorders/ or Neurodevelopmental disorders/ (48471)

4 exp Autism Spectrum Disorder/ (24730)

5 (anxiety or autism or autistic or "oppositional defiance" or "attention deficit*" or aggression or conduct disorder* or "obsessive compulsive disorder*" or neurodevelopmental disorder* or neuro-developmental disorder* or ADHD or ODD or ASD or OCD).ti,ab,kw,kf. (286907)

6 (behavio?r* adj3 (disorder* or problem* or issue*)).ti,ab,kw,kf. (42278)

7 1 or 2 or 3 or 4 or 5 or 6 (391713)

8 exp Serotonin Uptake Inhibitors/ (35340)

9 exp "Serotonin and Noradrenaline Reuptake Inhibitors"/ (3998)

10 exp Antipsychotic Agents/ (116226)

11 Atomoxetine hydrochloride/ (1088)

12 Clonidine/ (13042)

13 Imipramine/ (9834)

14 Guanfacine/ (660)

15 Melatonin/ (18285)

16 exp Central Nervous System Stimulants/ (92563)

17 (stimulant* or "serotonin uptake inhibitor*" or SSRI* or "serotonin noradrenaline reuptake inhibitor*" or "serotonin noradrenaline re-uptake inhibitor*" or SNRI* or antipsychotic* or anti-psychotic* or metylphenidate or clonidine or atomoxetine or imipramine or lamotrigine or fluoxetine or lisdexamfetamine or aripiprazole or guanfacine or melatonin or bumetanide or risperidone).ti,ab. (134752)

18 (psychopharmacotherap* or pharmacotherap*).ti,ab,kw,kf. (32812)

19 ((pharmacolog* or drug) adj2 (treatment* or therap*)).ti,ab,kw,kf. (142841)

20 8 or 9 or 10 or 11 or 12 or 13 or 14 or 15 or 16 or 17 or 18 or 19 (482642)

21 Urination disorders/ or exp Enuresis/ or exp Urinary incontinence/ or Urinary retention/ (48083)

22 bladder.ti,ab. (143777)

23 (void* adj1 (symptom* or dysfunction*)).ti,ab. (3804)

24 ((urinary or urine) adj5 (continence or incontinence or retention or retained or elimination or urgency)).ti,ab. (38908)

25 ((symptom* or dysfunction*) adj1 urinary tract).ti,ab. (9136)

26 enuresis.ti,ab,kw. (4822)

27 ((daytime or day time) adj (wetting or incontinence or voiding)).ti,ab,kw,kf. (359)

28 21 or 22 or 23 or 24 or 25 or 26 or 27 (197955)

29 (child or children or childhood or p?ediatric* or youth or adolescent* or adolescence or young people or young person* or infan*).ti,ab. (1796703)

30 adolescent/ or exp child/ or exp infant/ (3342664)

31 29 or 30 (3786454)

32 7 and 20 and 28 and 31 (140)

33 limit 32 to (english language and yr="1996 -Current") (71)

34 7 and 28 and 31 (1031)

35 limit 34 to (english language and yr="1996 -Current") (576)

36 20 and 28 and 31 (976)

37 limit 36 to (english language and yr="1996 -Current") (393)

38 37 not 35 (322)

39 35 or 38 (898)

Database: Embase <1947 to present>

Search Strategy:

--------------------------------------------------------------------------------

1 anxiety disorder/ (62046)

2 anxiety/ (188157)

3 behavior disorder/ (53872)

4 attention deficit disorder/ (53549)

5 exp autism/ (59412)

6 obsessive compulsive disorder/ (22226)

7 oppositional defiant disorder/ (2735)

8 conduct disorder/ (6682)

9 (anxiety or autism or autistic or oppositional defiance or attention deficit* or aggression or conduct disorder* or obsessive compulsive or ADHD or ODD or ASD or OCD or neuro-development* disorder* or neurodevelopment* disorder*).ti,ab. (399137)

10 (behavio?r* adj3 (disorder* or problem* or issue*)).ti,ab. (55513)

11 1 or 2 or 3 or 4 or 5 or 6 or 7 or 8 or 9 or 10 (564546)

12 exp serotonin uptake inhibitor/ (245796)

13 exp serotonin noradrenalin reuptake inhibitor/ (166147)

14 exp neuroleptic agent/ (278866)

15 exp central stimulant agent/ (294051)

16 atomoxetine/ (4925)

17 clonidine/ (40745)

18 guanfacine/ (3065)

19 melatonin/ (31949)

20 (stimulant* or "serotonin uptake inhibitor*" or SSRI* or "serotonin noradrenaline reuptake inhibitor*" or "serotonin noradrenaline re-uptake inhibitor*" or SNRI* or antipsychotic* or anti-psychotic* or metylphenidate or clonidine or atomoxetine or imipramine or lamotrigine or fluoxetine or lisdexamfetamine or aripiprazole or guanfacine or melatonin or bumetanide or risperidone).ti,ab. (189321)

21 (psychopharmacotherap* or pharmacotherap*).ti,ab. (49711)

22 ((pharmacolog* or drug) adj2 (treatment* or therap*)).ti,ab. (190524)

23 12 or 13 or 14 or 15 or 16 or 17 or 18 or 19 or 20 or 21 or 22 (949815)

24 exp urine incontinence/ (72492)

25 urinary tract function/ (1684)

26 exp bladder function/ (58824)

27 urine retention/ (26061)

28 micturition disorder/ (10063)

29 bladder.ti,ab. (216294)

30 (void* adj1 (symptom* or dysfunction*)).ti,ab. (6528)

31 ((urinary or urine) adj5 (continence or incontinence or retention or retained or elimination or urgency)).ti,ab. (63157)

32 ((symptom* or dysfunction*) adj1 urinary tract).ti,ab. (14565)

33 enuresis.ti,ab. (6673)

34 ((daytime or day time) adj (wetting or incontinence or voiding)).ti,ab. (483)

35 24 or 25 or 26 or 27 or 28 or 29 or 30 or 31 or 32 or 33 or 34 (330948)

36 exp child/ (2782916)

37 adolescent/ (1529478)

38 exp infant/ (1081679)

39 (child or children or childhood or youth or adolescent* or adolescence or young people or young person* or infan*).ti,ab. (2240921)

40 36 or 37 or 38 or 39 (4069383)

41 11 and 23 and 35 and 40 (702)

42 limit 41 to (english language and yr="1996 -Current") (585)

Annotation: Behaviour/drugs/bladder/kids all results contained in line 49

43 11 and 35 and 40 (2256)

44 limit 43 to (english language and yr="1996 -Current") (1666)

Annotation: Behaviour/bladder/kids

45 23 and 35 and 40 (2382)

46 limit 45 to (english language and yr="1996 -Current") (1471)

Annotation: Drugs/bladder/kids

47 44 or 46 (2552)

48 limit 47 to (books or chapter or conference abstract or conference paper or "conference review" or editorial or letter or note) (533)

49 47 not 48 (2019)

Annotation: Behaviour/bladder/kids OR Bladder/drugs/children

Database: PsycINFO <1806 to November Week 2 2021>

Search Strategy:

--------------------------------------------------------------------------------

1 anxiety disorders/ or obsessive compulsive disorder/ (29545)

2 anxiety/ (56806)

3 exp attention deficit disorder/ or oppositional defiant disorder/ (26001)

4 autism spectrum disorders/ (39156)

5 conduct disorder/ or aggressive behavior/ or behavior disorders/ (36362)

6 (anxiety or autism or autistic or oppositional defiance or attention deficit* or aggression or conduct disorder* or obsessive compulsive or ADHD or ODD or ASD or OCD or neuro-development* disorder* or neurodevelopment* disorder*).ti,ab. (306840)

7 1 or 2 or 3 or 4 or 5 or 6 (332400)

8 exp serotonin reuptake inhibitors/ (11863)

9 exp neuroleptic drugs/ (29816)

10 cns stimulating drugs/ (2820)

11 (stimulant* or "serotonin uptake inhibitor*" or SSRI* or "serotonin noradrenaline reuptake inhibitor*" or "serotonin noradrenaline re-uptake inhibitor*" or SNRI* or antipsychotic* or anti-psychotic* or metylphenidate or clonidine or atomoxetine or imipramine or lamotrigine or fluoxetine or lisdexamfetamine or aripiprazole or guanfacine or melatonin or bumetanide or risperidone).ti,ab. (62530)

12 (psychopharmacotherap* or pharmacotherap*).ti,ab. (13944)

13 ((pharmacolog* or drug) adj2 (treatment* or therap*)).ti,ab. (28270)

14 8 or 9 or 10 or 11 or 12 or 13 (110220)

15 urinary incontinence/ (1942)

16 urinary function disorders/ (566)

17 bladder/ (565)

18 bladder.ti,ab. (2317)

19 (void* adj1 (symptom* or dysfunction*)).ti,ab. (59)

20 ((urinary or urine) adj5 (continence or incontinence or retention or retained or elimination or urgency)).ti,ab. (1894)

21 ((symptom* or dysfunction*) adj1 urinary tract).ti,ab. (257)

22 enuresis.ti,ab. (1860)

23 ((daytime or day time) adj (wetting or incontinence or voiding)).ti,ab. (27)

24 15 or 16 or 17 or 18 or 19 or 20 or 21 or 22 or 23 (6257)

25 (child or children or childhood or p?ediatric* or youth or adolescent* or adolescence or young people or young person* or infan*).ti,ab. (842382)

26 7 and 14 and 24 and 25 (69)

27 limit 26 to (english language and yr="1996 -Current") (36)

28 7 and 24 and 25 (436)

29 limit 28 to (english language and yr="1996 -Current") (200)

30 14 and 24 and 25 (147)

31 limit 30 to (english language and yr="1996 -Current") (60)

32 29 or 31 (224)

33 limit 32 to (chapter or "column/opinion" or "comment/reply" or editorial or letter) (54)

34 32 not 33 (170)

35 limit 34 to "0200 book" (9)

36 34 not 35 (161)

Database: PsycINFO <1806 to November Week 2 2021>

Search Strategy:

--------------------------------------------------------------------------------

1 anxiety disorders/ or obsessive compulsive disorder/ (29545)

2 anxiety/ (56806)

3 exp attention deficit disorder/ or oppositional defiant disorder/ (26001)

4 autism spectrum disorders/ (39156)

5 conduct disorder/ or aggressive behavior/ or behavior disorders/ (36362)

6 (anxiety or autism or autistic or oppositional defiance or attention deficit* or aggression or conduct disorder* or obsessive compulsive or ADHD or ODD or ASD or OCD or neuro-development* disorder* or neurodevelopment* disorder*).ti,ab. (306840)

7 1 or 2 or 3 or 4 or 5 or 6 (332400)

8 exp serotonin reuptake inhibitors/ (11863)

9 exp neuroleptic drugs/ (29816)

10 cns stimulating drugs/ (2820)

11 (stimulant* or "serotonin uptake inhibitor*" or SSRI* or "serotonin noradrenaline reuptake inhibitor*" or "serotonin noradrenaline re-uptake inhibitor*" or SNRI* or antipsychotic* or anti-psychotic* or metylphenidate or clonidine or atomoxetine or imipramine or lamotrigine or fluoxetine or lisdexamfetamine or aripiprazole or guanfacine or melatonin or bumetanide or risperidone).ti,ab. (62530)

12 (psychopharmacotherap* or pharmacotherap*).ti,ab. (13944)

13 ((pharmacolog* or drug) adj2 (treatment* or therap*)).ti,ab. (28270)

14 8 or 9 or 10 or 11 or 12 or 13 (110220)

15 urinary incontinence/ (1942)

16 urinary function disorders/ (566)

17 bladder/ (565)

18 bladder.ti,ab. (2317)

19 (void* adj1 (symptom* or dysfunction*)).ti,ab. (59)

20 ((urinary or urine) adj5 (continence or incontinence or retention or retained or elimination or urgency)).ti,ab. (1894)

21 ((symptom* or dysfunction*) adj1 urinary tract).ti,ab. (257)

22 enuresis.ti,ab. (1860)

23 ((daytime or day time) adj (wetting or incontinence or voiding)).ti,ab. (27)

24 15 or 16 or 17 or 18 or 19 or 20 or 21 or 22 or 23 (6257)

25 (child or children or childhood or p?ediatric* or youth or adolescent* or adolescence or young people or young person* or infan*).ti,ab. (842382)

26 7 and 14 and 24 and 25 (69)

27 limit 26 to (english language and yr="1996 -Current") (36)

28 7 and 24 and 25 (436)

29 limit 28 to (english language and yr="1996 -Current") (200)

30 14 and 24 and 25 (147)

31 limit 30 to (english language and yr="1996 -Current") (60)

32 29 or 31 (224)

33 limit 32 to (chapter or "column/opinion" or "comment/reply" or editorial or letter) (54)

34 32 not 33 (170)

35 limit 34 to "0200 book" (9)

36 34 not 35 (161)

Search Name: Dilharan

Date Run: 15/11/2021 20:53:29

Comment: Cochrane

ID Search Hits

#1 MeSH descriptor: [Anxiety Disorders] explode all trees 5868

#2 MeSH descriptor: [Anxiety] this term only 6545

#3 MeSH descriptor: [Attention Deficit and Disruptive Behavior Disorders] explode all trees 2685

#4 MeSH descriptor: [Child Behavior Disorders] this term only 811

#5 MeSH descriptor: [Neurodevelopmental Disorders] this term only 61

#6 MeSH descriptor: [Autism Spectrum Disorder] explode all trees 1051

#7 (anxiety or autism or autistic or "oppositional defiance" or "attention deficit*" or aggression or "conduct disorder*" or "obsessive compulsive" or ADHD or ODD or ASD or OCD or "neuro-development* disorder*" or "neurodevelopment* disorder*"):ti,ab 38692

#8 #1 or #2 or #3 or #4 or #5 or #6 or #7 42449

#9 MeSH descriptor: [Serotonin Uptake Inhibitors] explode all trees 2599

#10 MeSH descriptor: [Serotonin and Noradrenaline Reuptake Inhibitors] explode all trees 26

#11 MeSH descriptor: [Antipsychotic Agents] explode all trees 4271

#12 MeSH descriptor: [Central Nervous System Stimulants] explode all trees 2178

#13 (stimulant* or "serotonin uptake inhibitor*" or SSRI* or "serotonin noradrenaline reuptake inhibitor*" or "serotonin noradrenaline re-uptake inhibitor*" or SNRI* or antipsychotic* or anti-psychotic* or metylphenidate or clonidine or atomoxetine or imipramine or lamotrigine or fluoxetine or lisdexamfetamine or aripiprazole or guanfacine or melatonin or bumetanide or risperidone):ti,ab 22157

#14 (psychopharmacotherap* or pharmacotherap*):ti,ab 4525

#15 ((pharmacolog* or drug) near/2 (treatment* or therap*)):ti,ab 16297

#16 #9 or #10 or #11 or #12 or #13 or #14 or #15 44932

#17 MeSH descriptor: [Urination Disorders] this term only 473

#18 MeSH descriptor: [Enuresis] explode all trees 462

#19 MeSH descriptor: [Urinary Incontinence] explode all trees 1895

#20 MeSH descriptor: [Urinary Retention] this term only 336

#21 bladder:ti,ab 8793

#22 (void* next (symptom* or dysfunction)):ti,ab 437

#23 ((urinary or urine) near/5 (continence or incontinence or retention or retained or elimination or urgency)):ti,ab 5876

#24 ((symptom* or dysfunction) next "urinary tract"):ti,ab 145

#25 enuresis:ti,ab 674

#26 ((daytime or day time) next (wetting or incontinence or voiding)):ti,ab 211

#27 #17 or #18 or #19 or #20 or #21 or #22 or #23 or #24 or #25 or #26 14550

#28 MeSH descriptor: [Child] explode all trees 1394

#29 MeSH descriptor: [Infant] explode all trees 15040

#30 MeSH descriptor: [Adolescent] explode all trees 97662

#31 (child or children or childhood or youth or adolescent* or adolescence or young people or young person* or infan* or p*ediatric*):ti,ab 130197

#32 #28 or #29 or #30 or #31 208934

#33 #8 and #16 and #27 and #32 14

#34 #8 and #27 and #32 54

#35 #27 and #16 and #32 163

#36 #34 or #35 with Cochrane Library publication date Between Jan 1996 and Nov 2021
