# Supplementary material for: Optimising the management of children with concomitant bladder dysfunction and behavioural disorders
Source: Eur Child Adolesc Psychiatry. 2022 Jun 29;32(10):1989–99. doi: 10.1007/s00787-022-02016-4 (PMC10533605; doi:10.1007/s00787-022-02016-4)
Supplement: Supplementary file 2 — Supplementary file2 (DOCX 35 kb) [file 787_2022_2016_MOESM2_ESM.docx]

pyscINFO

1806-2018

n = 149

PUBMED

1962-2018

n = 861

EMBASE

1996-2018

n = 978

MEDLINE

1996-2018

n = 298

Total number of identified through database searching

n = 2286

Records excluded based on inclusion/exclusion criteria

n = 1905

Total number of titles screened for eligibility

n = 2239

Total number of articles included for abstract review

n = 334

Duplicates removed

n = 59

Records excluded based on inclusion/exclusion criteria

n = 229

Number of articles for abstract review after duplicates removed

n = 275

Total number of articles for full text review

n = 46

Total number of articles included in systematic review

n = 46
